# Protective Effects of Currants (*Vitis vinifera*) on Corticolimbic Serotoninergic Alterations and Anxiety-like Comorbidity in a Rat Model of Parkinson’s Disease

**DOI:** 10.3390/ijms24010462

**Published:** 2022-12-27

**Authors:** Martha Tsarouchi, Eleni Fanarioti, Vaios T. Karathanos, Catherine R. Dermon

**Affiliations:** 1 Laboratory of Human and Animal Physiology, Department of Biology, University of Patras, 265 00 Patras, Greece; 2 Laboratory of Chemistry-Biochemistry-Physical Chemistry of Foods, Department of Dietetics and Nutrition, Harokopio University, 176 76 Kallithea, Greece; 3 Agricultural Cooperatives’ Union of Aeghion, Corinthou 201, 251 00 Aeghion, Greece

**Keywords:** Parkinson’s disease, serotonergic, noradrenergic, prefrontal cortices, basolateral amygdala, corticolimbic circuit

## Abstract

Parkinson’s disease (PD) is a progressive neurodegenerative disorder characterized by the loss of nigral dopaminergic neurons. Increasing evidence supports that PD is not simply a motor disorder but a systemic disease leading to motor and non-motor symptoms, including memory loss and neuropsychiatric conditions, with poor management of the non-motor deficits by the existing dopaminergic medication. Oxidative stress is considered a contributing factor for nigrostriatal degeneration, while antioxidant/anti-inflammatory properties of natural phyto-polyphenols have been suggested to have beneficial effects. The present study aimed to determine the contribution of monoaminergic neurotransmission on the anxiety-like phenotype in a rat rotenone PD model and evaluate the possible neuroprotective effects of black Corinthian currant, *Vitis vinifera*, consisting of antioxidant polyphenols. Rotenone-treated rats showed anxiety-like behavior and exploratory deficits, accompanied by changes in 5-HT, SERT and β_2_-ARs expression in the prefrontal cortices, hippocampus and basolateral amygdala. Importantly, the motor and non-motor behavior, as well as 5-HT, SERT and β_2_-ARs expression patterns of the PD-like phenotype were partially recovered by a supplementary diet with currants. Overall, our results suggest that the neuroprotective effects of Corinthian currants in rotenone-induced anxiety-like behavior may be mediated via corticolimbic serotonergic transmission.

## 1. Introduction

Parkinson’s disease (PD) represents the second most common neurodegenerative disorder, affecting up to ten million people worldwide, with a predicted increase of more than two-fold by 2030 [1]. A major neuropathological hallmark of PD is the degeneration and subsequent loss of dopaminergic neurons in the substantia nigra pars compacta (SNpc), resulting in a profound depletion of dopamine in striatal terminals that leads to clinical manifestations of resting tremor, bradykinesia, postural instability and rigidity. Increasing evidence suggests that PD is not simply a motor disorder but rather a disease with multiple non-motor symptoms, including neuropsychiatric and autonomic dysfunctions [2], occurring at early stages of PD [3]. Therapies currently available do not successfully address the non-motor manifestations, and studies on the underlying mechanisms would be of importance for early intervention strategies that would improve patients’ outcomes.

In PD, the neurodegenerative process extends beyond the dopaminergic system with extensive extra-striatal pathology, involving neurotransmitters such as serotonin (5-HT) and norepinephrine [4]. Moreover, a high incidence of depression and anxiety characterizes PD psychopathology [5] suggesting a complex dysfunction of noradrenergic and serotonergic systems [2,6]. Indeed, loss of noradrenaline terminals in PD patients [7] contributes to the anxiety symptoms of PD, in line with evidence implicating the NAergic system in emotional processing [8]. On the other hand, serotonin has long been implicated in the pathophysiology of psychiatric disorders. In accordance with this, neuroimaging studies relay a high prevalence of anxiety and PD severity with complex functional connectivity changes in limbic areas, including the basolateral amygdala (BLA), hippocampus and prefrontal cortex (PFC) [9], which are the main targets of NAergic and 5-HT neurons [10].

The higher susceptibility of dopaminergic neurons to mitochondrial oxidative stress possibly leads to impairment of the dopamine metabolism and degeneration of substantia nigra (SNpc) tyrosine hydroxylase (TH) neurons in PD. Polyphenols are suggested as acting as potent antioxidants against PD neurodegeneration by up-regulating mitochondrial complex-I activity and preventing dopaminergic neuronal cell loss [11], and have been shown to exert neuroprotective effects in animal models, reducing oxidative stress and neuroinflammation [12]. Polar phenolics can cross the blood–brain barrier and accumulate in distinct brain regions such as the striatum, mesencephalon, hippocampus, frontal cortex and cerebellum [13,14,15]. Increasing evidence suggests that diet and dietary components might significantly reduce the risk or alleviate the severity of PD [16,17]. In particular, grape seeds containing a mixture of polyphenolic flavonoids are associated with a wide range of biological functions, including anti-inflammatory [18], anti-mutagenic, anti-carcinogenic and anti-apoptotic [19,20] events.

In the present study, a neurotoxic-rotenone rat model was used to produce a PD-like phenotype [21] and anxiety-like comorbidity. Rotenone, a naturally occurring pesticide, crosses the blood–brain barrier, causing neurotoxicity, specifically inhibiting mitochondrial complex-I, and induces the selective degeneration of the nigrostriatal dopaminergic neurons, resulting in neurochemical and behavioral PD-like features [22,23]. In agreement with this, we have previously shown that rotenone administration impaired locomotor activity and decreased TH and BDNF expression in the rat nigrostriatal pathway, exhibiting parkinsonian-like features [21]. Based on the consensus that fundamental neurophysiological mechanisms are conserved across species, we questioned whether corticolimbic noradrenergic and serotonergic changes underlie the anxiety-like comorbidity in PD and whether a Corinthian currant supplemental diet can partly recover the behavioral and neurochemical profile using a rat model of rotenone-induced PD phenotype.

## 2. Results

### 2.1. Effects of Rotenone Treatment and Currant Consumption on Thigmotaxic Behavior Using the Open Field Test (OFT)

**Figure 1:**
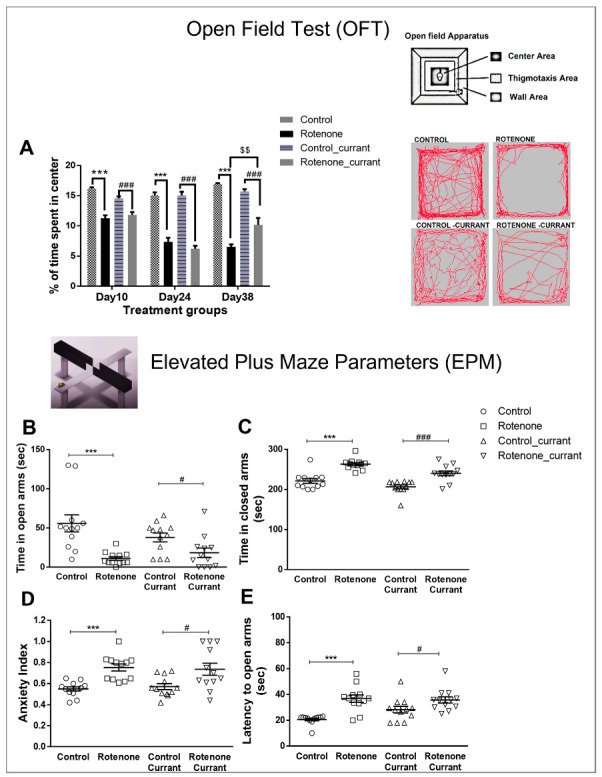
Anxiety-like phenotype in a rotenone-induced PD-like model with or without currant consumption. (**A**) Thigmotactic responses based on the time spent in the center of the OFT and time spent in the center area (as a percentage of total time). Representative track plot tracings generated by Ethovision software demonstrate ambulatory activity in controls and rotenone-treated rats with or without currant consumption. (**B**–**E**) Anxiety-like phenotype estimated by Elevated Plus-Maze (EPM). Time spent in the EPM open (**B**) and closed (**C**) arms. (**D**) Anxiety Index. (**E**) Latency to first entry in the EPM open arm. Data are expressed as mean ± SEM, n = 12/group. *** *p* ≤ 0.001 (Control/Rotenone), # *p* ≤ 0.05, ### *p* ≤ 0.001 (Control_currant/Rotenone_currant), $$ *p* ≤ 0.01, (Rotenone/Rotenone_currant).

We used the OFT to evaluate the exploratory behavior of new environments and the innate fear in rats of open spaces that trigger anxiety. Their preference to remain longer in the periphery of the OFT in comparison to the central areas is considered a thigmotaxic response, and this was used as an index of anxiety in rats treated with vehicle and rotenone with or without a complementary diet with currants (Figure 1A). Two-way repeated measures ANOVA indicated significant effects of treatment (F(1.44) = 239.848, *p* < 0.001) and interaction (treatment × currant, F(1.44) = 5.425, *p* = 0.025) but not a significant currant (F(1.44) = 0.04, *p* = 0.953) effect. Specifically, the duration of visits to the center zone of the OFT (during a period of 10 min) was significantly decreased in rotenone-treated rats (*p* < 0.001). In line with this, rotenone treatment with or without currant consumption resulted in a statistically significant reduction in the duration of the visits to the center zone of the OFT, when measured at 10, 24 and 38 (*p* < 0.001) days. Overall, thigmotaxis, the preference to the periphery and walls, was increased in rotenone-treated rats, as indicated by the reduced time spent in the center zone. Importantly, at day 38, a statistically significant decrease in thigmotaxis was observed in the rotenone group who consumed currants (*p* = 0.006) when compared with the rotenone group who consumed conventional food (Figure 1A).

### 2.2. Effects of Rotenone Treatment and Currant Consumption on Behavioral Parameters in the Elevated Plus-Maze (EPM)

The EPM is a widely used behavioral assay for assessing the anxiety responses of rodents. To determine whether a supplementary diet with currant improved the anxiety-like behavior of rotenone-treated rats, on day 38, rats were subjected to the elevated plus-maze test (Figure 1B–D). Rotenone-treated animals showed significantly higher levels of anxiety-like behavior. Considering the time animals spent in open arms, two-way ANOVA indicated a significant effect of treatment (F(1.47) = 21.650, *p* < 0.001) but not of currant consumption (F(1.47) = 0.583, *p* = 0.449) nor interaction (treatment × currant, F(1.47) = 3.368, *p* = 0.073). Thus, the duration of visits to the open arms of the EPM (during a period of 5 min) was significantly decreased in rotenone-treated rats with (*p* = 0.027) or without (*p* = 0.001) currant intake (Figure 1B). In line with this result, considering the duration of visits in closed arms, two-way ANOVA demonstrated significant effects of treatment (F(1.47) = 52.969, *p* < 0.001) and currant consumption (F(1.47) = 12.935, *p* = 0.001) but not significant interaction (treatment × currant, F(1.47) = 0.614, *p* = 0.437) (Figure 1C). An independent *t*-Test revealed a significantly increased duration of visits in closed arms of both rotenone (*p* < 0.001), and rotenone-currant (*p* < 0.001) groups indicating their higher levels of anxiety. For the anxiety index, two-way ANOVA revealed a significant effect of treatment (F(1.47) = 24.334, *p* < 0.001) but not of currant consumption (F(1.47) = 0.008, *p* = 0.929) nor interaction (treatment × currant, F(1.47) = 0.264, *p* = 0.610). Overall, the anxiety index, calculated based on the number of visits and duration in open and closed arms was different in the four groups of rats with higher values in the rotenone-treated rats with (*p* = 0.016) or without (*p* < 0.001) currant intake (Figure 1D). The latency to the first entry in the open arms was significantly prolonged in the rotenone group with (*p* = 0.046) or without (*p* < 0.001) a currant diet compared to the equivalent control group (Figure 1E), with a statistically significant effect of treatment (F(1.47) = 25.382, *p* < 0.001) but not of currant consumption (F(1.47) = 1.897, *p* = 0.175) nor interaction (treatment × currant, F(1.47) = 3.299, *p* = 0.076).

### 2.3. Prefrontal Cortex, Hippocampus and Basolateral Amygdala 5-HT Immunoreactivity Following Rotenone Treatment and Complementary Diet with Currants

**Figure 2:**
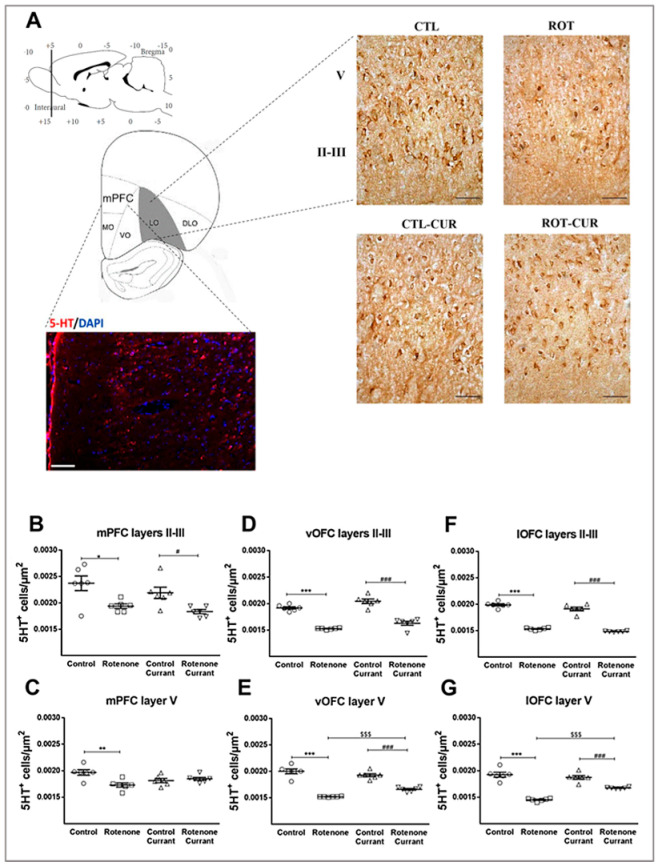
Altered 5-HT immunodensity across the prefrontal network in a rotenone-induced PD-like model with or without currant consumption. (**A**) Representative photomicrographs of lOFC sections immunostained for 5-HT are presented, scale bar = 0.05 mm. Co-labeling of 5-HT positive cells (red) with nuclear marker DAPI (blue) in mPFC of control rats indicating that serotonin labeling is cytoplasmic, scale bar = 0.1 mm. Quantification of 5-HT immunoreactivity in mPFC layers (**B**) II–III and (**C**) V, vOFC layers (**D**) II–III and (**E**) V, (**C**) lOFC layers (**F**) II–III and (**G**) V. Schematic diagram is based on the rat brain atlas of Paxinos and Watson [24] indicating the locus of the mPFC, vOFC and lOFC. Data are expressed as mean ± SEM, n = 6 per group. * *p* ≤ 0.05, ** *p* ≤ 0.01, *** *p* ≤ 0.001 (Control vs. Rotenone), # *p* ≤ 0.05, ### *p* ≤ 0.001 (Control_currant vs. Rotenone_currant), $$$ *p* ≤ 0.001 (Rotenone vs. Rotenone_currant). mPFC: medial Prefrontal cortex, vOFC: ventral Orbital Frontal cortex, lOFC: lateral Orbital frontal cortex.

***Prefrontal cortices:*** The rat medial prefrontal cortex (mPFC), located at anteroposterior (AP) +4.68 to +3.50 mm from bregma, mediolateral (ML) ± 0.7 mm from midline and dorsoventral (DV) −3.5 mm from dura, is also called the prelimbic cortex (PrL). The mPFC contains layer I, layer II/III (associative layers) and layer V/VI (output layers). mPFC layers II/III 5-HT^+^ cell density showed a significant simple main effect of rotenone treatment (F(1.23) = 18.159, *p* < 0.001), but no significant currant consumption (F(1.23) = 2.380, *p* = 0.139) nor interaction (F(1.23) = 0.177, *p* = 0.678) effects were indicated. Specifically, a reduction in 5-HT^+^ cells in mPFC layers II/III of the rotenone-treated (*p* = 0.014) and rotenone-currant (*p* = 0.011) groups was observed compared to the equivalent controls (Figure 2B and Supplementary Figure S1). In the output layer V of mPFC, two-way ANOVA revealed a significant interaction of rotenone treatment × currant consumption (F(1.23) = 10.955, *p* = 0.003), with significant simple main effect of rotenone treatment (F(1.23) = 6.377, *p* = 0.020) but there was no difference between the groups consuming currants (F(1.23) = 0.174, *p* = 0.681). Rotenone treatment significantly decreased layer V 5-HT^+^ cell density (*p* = 0.004) compared to the control group (Figure 2C and Supplementary Figure S1).

Subsequently, we examined the effects of rotenone and black Corinthian currant consumption on 5-HT^+^ cell density in rat ventral and lateral orbitofrontal cortexes (vOFC, lOFC), located at anteroposterior (AP) +4.68 to +3.50 mm from bregma. No significant interaction between rotenone treatment and supplementary nutrition with currants in layers II/III of vOFC (F(1.23) = 0.120, *p* = 0.732) and lOFC (F(1.23) = 0.365, *p* = 0.553) was determined. However, rotenone treatment resulted in a significant simple main effect in layers II/III of vOFC (F(1.23) = 167.546, *p* < 0.001) (Figure 2D and Supplementary Figure S2) and lOFC (F(1.23) = 469.081, *p* < 0.001) (Figure 2F), showing a reduction in 5-HT^+^ cells of rotenone-treated rats (*p* < 0.001) with and without currant intake compared to their controls. In contrast, a significant interaction of rotenone treatment × currant consumption characterized layer V of vOFC (F(1.23) = 14.322, *p* = 0.001) and lOFC (F(1.23) = 22.376, *p* < 0.001), with a significant simple main effect of rotenone treatment (vOFC: F(1.23) = 175.441, *p* < 0.001, lOFC: F(1.23) = 134.814, *p* < 0.001), showed reduced 5-HT^+^ cell density in the rotenone-treated group (*p* < 0.001). Importantly, no significant difference between the groups that consumed currants (F(1.23) = 1.343, *p* = 0.260) was found for vOFC. A small but significant recovery of vOFC (Figure 2E and Supplementary Figure S2) and lOFC (Figure 2G) layer V 5-HT^+^ cells was detected in the rotenone-currant-treated group compared to the rotenone group consuming conventional food (*p* < 0.001). In addition, lOFC layer V showed a statistically significant currant effect (F(1.23) = 8.856, *p* = 0.007).

**Figure 3:**
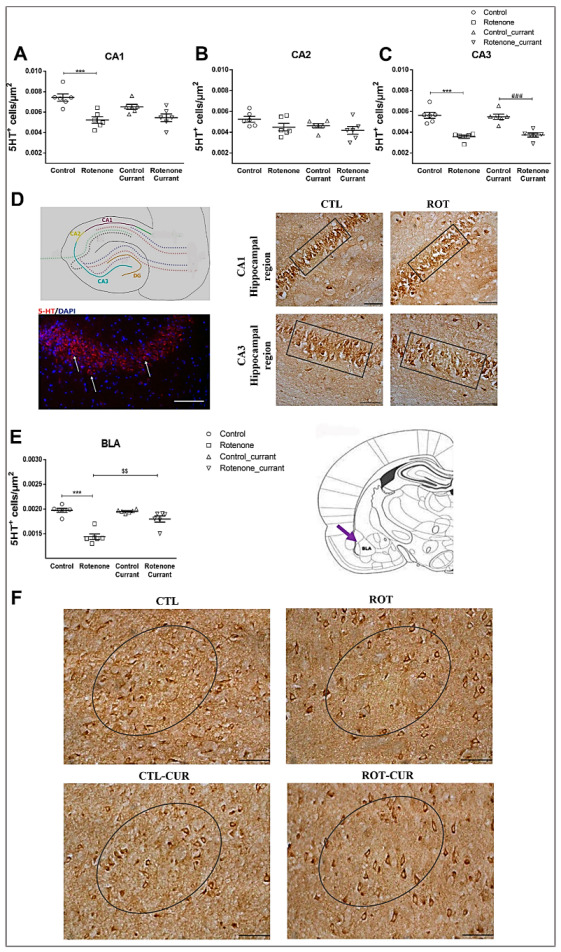
Altered 5-HT immunodensity in the hippocampus and BLA in a rotenone-induced PD model with or without currant consumption. Quantification of 5-HT immunoreactivity in (**A**–**C**) the hippocampus. (**D**) Representative photomicrographs of hippocampal sections immunostained for 5-HT (scale bar = 0.05 mm) at the coronal level shown in the schematic diagram and co-labeling of 5-HT positive cells (red) with nuclear marker DAPI (blue) in CA2 the hippocampal region of control rats, depicting serotonin labeling is cytoplasmic (scale bar = 0.1 mm). CA1 and CA3 areas of interest are shown in boxes. (**E**) Quantification of 5-HT immunoreactivity in BLA. (**F**) Representative photomicrographs of BLA sections immunostained for 5-HT at the coronal level shown in the schematic diagram based on rat brain atlas of Paxinos and Watson [24]. BLA areas of interest are included in circles. Scale bar = 0.05 mm. Data are expressed as mean ± SEM, n = 6 per group. *** *p* ≤ 0.001 (Control vs. Rotenone), ### *p* ≤ 0.001 (Control_currant vs. Rotenone_currant), $$ *p* ≤ 0.01, (Rotenone vs. Rotenone_currant). BLA: Basolateral Amygdala.

***Hippocampus:*** Two-way ANOVA indicated no statistically significant interaction between the effects of rotenone treatment and currant consumption in CA1 (F(1.23) = 2.997, *p* = 0.099) and CA3 (F(1.23) = 0.474, *p* = 0.499) hippocampal regions. Rotenone treatment caused a significant simple main effect in 5-HT^+^ cell density of CA1 (F(1.23) = 24.818, *p* < 0.001) and CA3 (F(1.23) = 68.438, *p* < 0.001) and particularly a reduction in 5-HT^+^ cells of rotenone-treated rats (*p* = 0.001) without currant intake compared to their controls, whereas no significant effect was observed for currant consumption in CA1 (F(1.23) = 1.120, *p* = 0.303) (Figure 3A) and CA3 (F(1.23) = 0.005, *p* = 0.942) (Figure 3C). In contrast to CA1 and CA3, CA2 did not show a significant simple main effect of rotenone treatment (F(1.23) = 3.747, *p* = 0.067) or currant intake (F(1.23) = 2.292, *p* = 0.146) (Figure 3B), nor an interaction between the effects of rotenone treatment and currant consumption (F(1.23) = 0.276, *p* = 0.605).

***Amygdaloid Complex*:** Interestingly, 5-HT^+^ cell density in BLA showed a significant interaction between rotenone treatment and currant administration (F(1.23) = 16.209, *p* = 0.001), with a statistically significant simple main effect of rotenone treatment (F(1.23) = 53.871, *p* < 0.001) and currant consumption (F(1.23) = 12.787, *p* = 0.002) (Figure 3E). Decreased 5-HT^+^ cell densities were observed in the rotenone group without currant intake compared to their controls (*p* < 0.001). A slight but significant recovery of 5-HT^+^ cells was observed in the BLA of the rotenone-treated group who consumed currants compared with the rotenone group consuming conventional food (*p* = 0.002).

### 2.4. SERT Expression Pattern Following Rotenone Treatment and Complementary Diet with Currants

**Figure 4:**
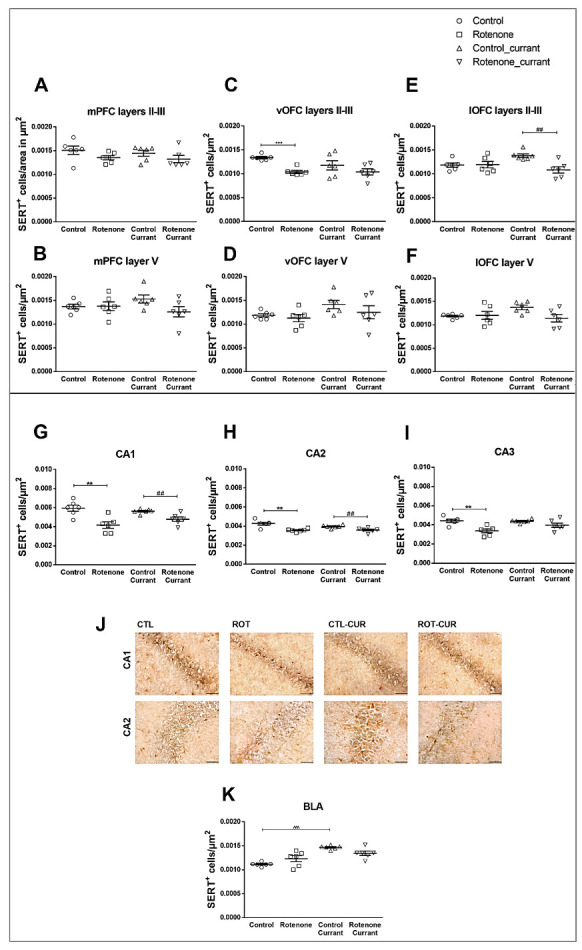
Altered SERT immunodensity in a rotenone-induced PD model with or without currant consumption. Quantification of SERT immunoreactivity in mPFC layers (**A**) II–III and (**B**) V, vOFC layers (**C**) II–III and (**D**) V, lOFC layers (**E**) II–III and (**F**) V, (**G**) CA1, (**H**) CA2, (**I**) CA3 hippocampal regions and (**K**) BLA. Representative photomicrographs of hippocampal CA1 and CA2 sections immunostained for SERT are shown in (**J**). Scale bar = 0.05 mm. Data are expressed as mean ± SEM, n = 6 per group. ** *p* ≤ 0.01, *** *p* ≤ 0.001 (Control vs. Rotenone), ## *p* ≤ 0.01 (Control_currant vs. Rotenone_currant), ^^^ *p* ≤ 0.001 (Control vs. Control_currant).

***Prefrontal Cortices:*** Two-way ANOVA indicated no significant effects on SERT immunodensity in mPFC layers II/III with treatment (F(1.23) = 4.068, *p* = 0.057), currant intake (F(1.23) = 0.422, *p* = 0.523) or interaction (F(1.23) = 0.066, *p* = 0.800) (Figure 4A and Supplementary Figure S3A). Similarly, no significant effects were found in mPFC layer V with treatment (F(1.23) = 2.329, *p* = 0.143), currant intake (F(1.23) = 0.065, *p* = 0.802) or interaction (F(1.23) = 2.620, *p* = 0.121) (Figure 4B and Supplementary Figure S3A). In vOFC layers II/III, rotenone treatment showed a significant simple main effect (F(1.23) = 12.722, *p* = 0.002) with a reduction in the SERT^+^ cells of rotenone-treated rats (*p* < 0.001) compared to controls (Figure 4C). SERT^+^ cell density in the lateral part of OFC (lOFC) layers II/III showed a significant interaction with rotenone treatment and currant consumption (F(1.23) = 7.596, *p* = 0.012). Rotenone treatment resulted in a significant simple main effect (F(1.23) = 6.558, *p* = 0.019) (Figure 4E), but no significant currant consumption effect (F(1.23) = 0.646, *p* = 0.431). No significant effects were found in layers V of vOFC with treatment (F(1.23) = 1.544, *p* = 0.228), currant intake (F(1.23) = 3.771, *p* = 0.066) or interaction (F(1.23) = 0.336, *p* = 0.569) (Figure 4D) and of lOFC with treatment (F(1.23) = 2.795, *p* = 0.110), currant intake (F(1.23) = 1.030, *p* = 0.322) or interaction (F(1.23) = 3.845, *p* = 0.064) (Figure 4F).

***Hippocampus:*** Two-way ANOVA indicated no significant interaction between the effects of rotenone treatment and currant administration in CA1 (F(1.23) = 3.076, *p* = 0.095), CA2 (F(1.23) = 2.598, *p* = 0.123) and CA3 (F(1.23) = 3.357, *p* = 0.082). Rotenone treatment caused a significant simple main effect in CA1 (F(1.23) = 24.222, *p* < 0.001) and CA2 (F(1.23) = 28.549, *p* < 0.001) with a reduction in SERT^+^ cells in rotenone-treated rats with (*p* = 0.008 for CA1 and *p* = 0.009 for CA2) or without (*p* = 0.003 for CA1 and *p* = 0.002 for CA2) currant intake compared to their controls (Figure 4G,H). Rotenone treatment caused a simple main effect in CA3 SERT immunodensity (F(1.23) = 17.527, *p* < 0.001), with decreases in the rotenone-treated group (*p* = 0.002) compared to the controls (Figure 4I). Νo statistically significant effects of currant consumption were found in CA1 (F(1.23) = 0.347, *p* = 0.563), CA2 (F(1.23) = 1.958, *p* = 0.177) and CA3 (F(1.23) = 2.513, *p* = 0.129).

**Figure 5:**
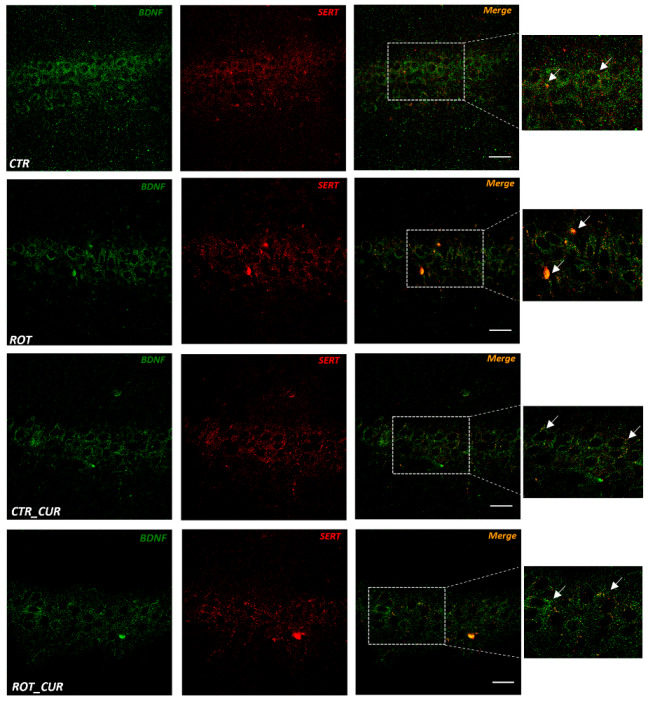
Indicative confocal immunofluorescent microphotographs showing the colocalization of SERT^+^ and BDNF^+^ cells in CA1 of control and rotenone-treated rats with and without currant consumption. A population of SERT^+^ (red) cells were also found to express BDNF (green) in CA1. Arrows show indicative double-labelled cells of SERT^+^ and BDNF^+^. Scale bar = 0.025 mm.

Next, we questioned whether the BDNF neurotrophic factor is colocalized in SERT expressing cells since it has been suggested to have a role in the pathogenesis of neurodegenerative conditions and mood-related behaviors. Interestingly, a population of hippocampal SERT immunopositive neurons (Figure 5) also expressed BDNF, highlighting its potential to influence the serotonergic function.

***Amygdaloid Complex:*** SERT^+^ cell density in BLA showed a significant interaction of rotenone treatment × currant consumption (F(1.23) = 8.738, *p* = 0.008) and significant simple main effect of currant consumption (F(1.23) = 32.565, *p* < 0.001), but no significant effect of rotenone treatment (F(1.23) = 0.015, *p* = 0.903). Further analysis with the independent T-test revealed a significant increase in the control group consuming currants compared to control group (*p* < 0.001) (Figure 4K).

### 2.5. β_2_-AR Expression Pattern Following Rotenone Treatment and Complementary Diet with Currants

**Figure 6:**
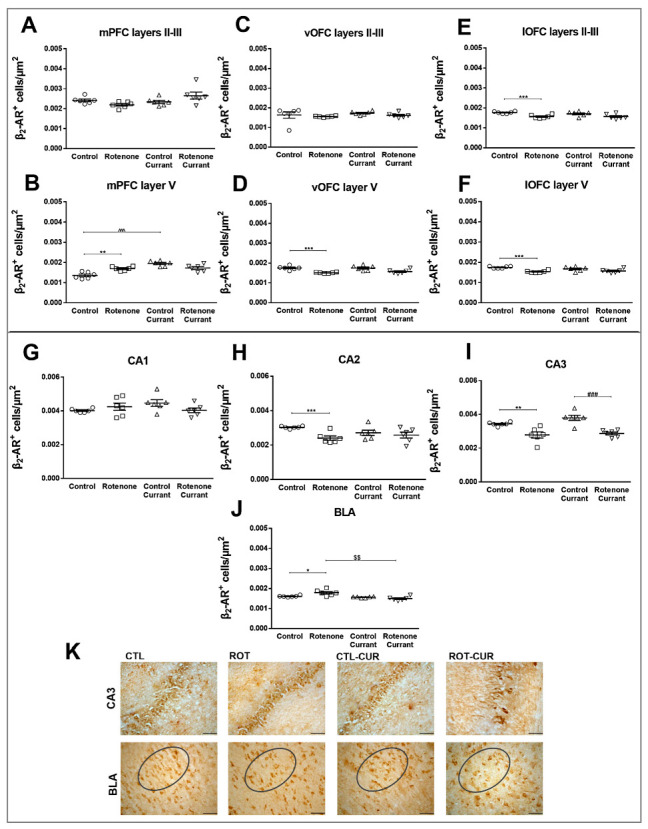
Altered β_2_-ARs imunodensity in a rotenone-induced PD model with or without currant consumption. Quantitative β_2_-ARs immunoreactivity in mPFC layers (**A**) II–III and (**B**) V, vOFC layers (**C**) II–III and (**D**) V, lOFC layers (**E**) II–III and (**F**) V, (**G**) CA1, (**H**) CA2, (**I**) CA3 hippocampal regions and (**J**) BLA. (**K**) Representative photomicrographs of areas of interest of hippocampal CA3 and BLA (circles) immunostaining for β_2_-ARs in all groups studied. Scale bar = 0.05 mm. Data are expressed as mean ± SEM, n = 6 per group. * *p* ≤ 0.05, ** *p* ≤ 0.01, *** *p* ≤ 0.001 (Control vs. Rotenone), ### *p* ≤ 0.001 (Control_currant vs. Rotenone_currant), ^$$^ *p* ≤ 0.01 (Rotenone vs. Rotenone_currant), ^^^ *p* ≤ 0.001 (Control vs. Control_currant).

***Prefrontal Cortices****:* The β_2_-AR^+^ cell density in mPFC showed a significant interaction of rotenone treatment × currant consumption in layers II/III (F(1.23) = 6.309, *p* = 0.021) and layer V (F(1.23) = 22.683, *p* < 0.001). No simple main effects of rotenone treatment were found in mPFC layers II/III (F(1.23) = 0.189, *p* = 0.668) or layer V (F(1,23) = 0.875, *p* = 0.361). Specifically, layers II/III did not show any significant effects of supplementary nutrition with currants (F(1.23) = 3.234, *p* = 0.087) (Figure 6A and Supplementary Figure S3B), in contrast to layer V, which showed a significant effect between the groups consuming currants (F(1.23) = 28.103, *p* < 0.001). Furthermore, a significant increase in β_2_-AR^+^ cells in layer V of mPFC of the rotenone-treated group was observed (*p* = 0.002) compared to the control group, as well as between the control-currant group compared to the control group consuming conventional food (*p* < 0.001) (Figure 6B and Supplementary Figure S3B).

In vOFC, β_2_-AR immunodensity did not show any significant interaction between the rotenone treatment and currant consumption in layers II/III (F(1.23) = 0.038, *p* = 0.848) or layer V (F(1.23) = 0.616, *p* = 0.442). In vOFC layers II/III, the simple main effects of rotenone treatment (F(1.23) = 1.249, *p* = 0.277) and currant intake (F(1.23) = 0.830, *p* = 0.373) were not statistically significant, but in vOFC layer V, rotenone treatment showed a significant simple main effect (F(1.23) = 26.432, *p* < 0.001) resulting in a reduction in β_2_-AR^+^ cells in the rotenone group (*p* < 0.001) compared to their matching controls. In contrast, the simple main effect of currant consumption (F(1.23) = 0.637, *p* = 0.434) was not significant (Figure 6C,D).

Similarly, no statistically significant interaction between the effects of rotenone and black Corinthian currant consumption on β_2_-AR^+^ cell density were detected in the lateral OFC layers II/III (F(1.23) = 0.605, *p* = 0.446) or layer V (F(1.23) = 2.510, *p* = 0.129). Rotenone treatment caused a significant simple main effect in lOFC II/III (F(1.23) = 17.133, *p* = 0.001) and lOFC layer V (F(1.23) = 21.211, *p* < 0.001). The simple main effect of currant intake was not statistically significant in layers II/III (F(1.23) = 0.380, *p* = 0.545) or layer V (F(1.23) = 0.229, *p* = 0.637). Further analysis with the independent T-test revealed a significant decrease in β_2_-AR+ cells in all layers studied in rotenone-treated rats compared to their controls (*p* < 0.001) (Figure 6E,F).

***Hippocampus:*** CA1 β_2_-AR immunodensity showed no statistically significant interaction between rotenone treatment and currant consumption (F(1.23) = 4.053, *p* = 0.058) as well as for the simple main effects of rotenone treatment (F(1.23) = 0.365, *p* = 0.553) and currant intake (F(1.23) = 0.496, *p* = 0.489) (Figure 6G). Similarly, CA3 β_2_-AR immunodensity showed no statistically significant interaction (F(1.23) = 1.122, *p* = 0.302) as well as for the simple main effect of currant intake (F(1.23) = 3.071, *p* = 0.095). Notably, two-way ANOVA indicated that rotenone treatment caused a significant simple main effect (F(1.23) = 37.944, *p* < 0.001) and particularly a reduction in β_2_-AR immunodensity of the rotenone-treated groups with (*p* < 0.001) or without (*p* = 0.005) a currant diet compared to their matching controls (Figure 6I). In CA2, β_2_-AR^+^ cell density showed a significant simple main effect of rotenone treatment (F(1.23) = 8.558, *p* = 0.008), but no significant currant intake (F(1.23) = 0.336, *p* = 0.568) or interaction (F(1.23) = 3.638, *p* = 0.071) effects. Further analysis with the independent T-test revealed a significant decrease in β_2_-AR immunodensity of rotenone-treated rats without a currant diet (*p* = 0.005) compared to their controls (Figure 6H).

***Amygdaloid Complex:*** Two-way ANOVA indicated a significant interaction between the effects of rotenone treatment and currant consumption (F(1.23) = 10.651, *p* = 0.004). Similarly, the simple main effect of currant consumption (F(1.23) = 19.701, *p* < 0.001) was significant but not of rotenone treatment (F(1.23) = 2.062, *p* = 0.166). A significant increase in β_2_-AR^+^ cells was observed in the rotenone group (*p* = 0.017) compared to controls. Decreased β_2_-AR^+^ cell density was observed in the BLA of the rotenone-currant group compared with the rotenone group (*p* = 0.002) (Figure 6J).

### 2.6. Anxiety-like Behavior Is Correlated with Altered 5-HT and SERT Immunodensity Levels

**Figure 7:**
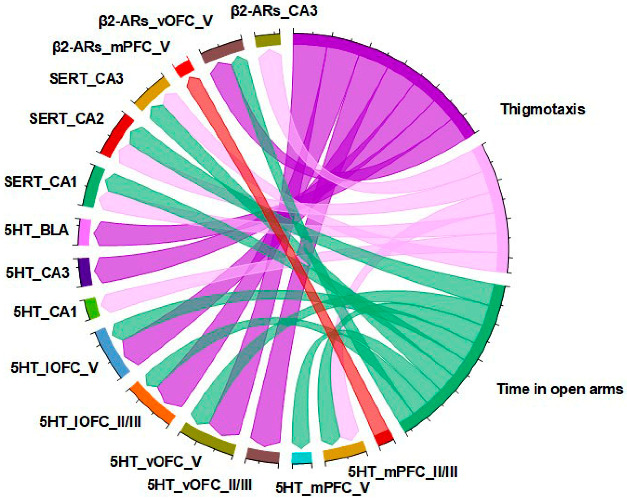
Chord diagram obtained from Pearson correlation analysis between neurochemical alterations and behavioral tests for anxiety. Links indicate strong positive (dark purple) correlations with “r” values above 0.65, moderate negative (red) correlations (“r” values below −0.42) and positive (light purple and green) correlations with “r” values between 0.47 and 0.64.

Pearson product-moment correlation analysis determined the possible relationship between neurochemical alterations and behavioral tests for anxiety (Figure 7). We examined whether the observed changes of 5-HT, SERT and β_2_-ARs expressing cells across the cortico-limbic system were correlated with the anxiety-like parameters. Our results indicated strong positive correlations specifically between the density of 5-HT expressing cells in mPFC layers II/III (r = 0.561, *p* = 0.01), vOFC layers II/III (r = 0.843, *p* < 0.001) and V (r = 0.909, *p* < 0.001), lOFC layers II/III (r = 0.898, *p* < 0.001) and V (r = 0.828, *p* < 0.001), as well as CA1 (r = 0.523, *p* = 0.018) and CA3 hippocampal regions (r = 0.723, *p* < 0.001) with the time spent in the center of the OFT. In addition, a positive correlation between the density of SERT expressing cells in CA1 (r = 0.553, *p* = 0.011), CA2 (r = 0.603, *p* = 0.005) and CA3 (r = 0.510, *p* = 0.022) hippocampal regions and the time spent in the center of the OFT was determined. Moreover, β_2_-AR density in vOFC layer V (r = 0,659, *p* < 0.001) as well as the CA3 (r = 0.636, *p* < 0.001) hippocampal region were positively correlated with the time spent in the center of the OFT.

We further examined whether the observed neurochemical changes across the cortico-limbic system are correlated with time spent in the open arms of the EPM. Our results indicate that the density of 5-HT expressing cells in mPFC layers II/II (r = 0.528, *p* = 0.017) and V (r = 0.499, *p* = 0.025), vOFC layer V (r = 0.566, *p* = 0.009), lOFC layers II/II (r = 0.473, *p* = 0.035) and V (r = 0.523, *p* = 0.018), as well as the density of SERT expressing cells in CA1 (r = 0.519, *p* = 0.019), CA2 (r = 0.594, *p* = 0.006) and CA3 (r = 0.517, *p* = 0.020) hippocampal regions were positively correlated with the time spent in the open arms of the EPM. In addition, the time spent in the open arms of the EPM was positively correlated (r = 0.452, *p* = 0.027) with the density of β_2_-ARs expressing cells in vOFC layer V but negatively correlated (r = −0.421, *p* = 0.040) with ARs in mPFC layer V.

## 3. Discussion

Induction of PD models using neurotoxins is a valuable tool for investigating the molecular basis of the disease and testing new potential therapeutic strategies. Rotenone treatment results in the loss of TH expressing cells in the SNpc, an underlying factor for the development of motor dysfunction in PD [21,25]. In addition to dopaminergic disruptions, noradrenergic and 5-HT monoamine systems have been suggested to be involved in mechanisms of PD pathogenesis. In line with this, the present study continuing our previous work [21], showed that the dopaminergic nigrostriatal dysfunction in the rat rotenone model was accompanied with an anxiety-like phenotype, associated with basolateral amygdala, hippocampus and medial and orbitofrontal prefrontal cortices 5-HT and noradrenergic neurotransmission. Importantly, a partial recovery of these behavioral and monoaminergic dysfunctions was evident following 38 days of black Corinthian currant consumption, a food source rich in polyphenols. Further identification of monoaminergic alterations and their modulation by dietary natural polyphenols could contribute to our understanding of the manifestation of anxiety/depression in PD, and possibly provide novel complementary intervention approaches.

Our previous study showed that rotenone-treated rats exhibited impaired locomotor activity with reduced velocity, distance traveled and increased immobility time in the OFT. Moreover, it has been documented that these motor deficits were accompanied by significant reductions in TH expressing neurons in both subdivisions of SNpc (lateral and medial) and significant decreases in TH immunodensities (ROD) and TH protein levels in the striatum [21] that includes the SNpc dopaminergic terminals. In addition to motor impairment, rotenone-treated rats exhibited anxiety-like behavior in OFT and EPM. Thigmotaxis [26] and anxiogenic-like effects [27] have been previously validated following 6-OHDA lesions in rats, but not in mice MPTP lesions [28]. The present data are in line with the frequent comorbidity of anxiety/depression observed in PD patients [29], supporting that the manifestation of anxiety can be successfully modeled in the rotenone PD rat model.

Monoaminergic systems have a crucial modulatory role in the organization of corticolimbic circuits of the PFC, hippocampus and amygdala, which are known to process goal-directed behavior, emotions and cognitive information [30,31] and have been suggested to contribute to the anxiety accompanying PD [9]. In accordance, noradrenergic and serotonergic transmission is modulated by the dopaminergic deficiency in PD motor pathology [6]. Indeed, serotonergic and noradrenergic signaling are well associated with emotional responses, especially those of fear and anxiety [32]. The present data clearly showed a rotenone-induced decreased serotonergic immunoreactivity in the corticolimbic circuit, in agreement with previous reports on the degeneration of the 5-HT transmission system [33] and decreased serotonin concentrations in the PFC in PD [34], as well as with evidence that selective serotonin reuptake inhibitors can help the depressive symptomatology in PD [35]. Indeed, increased anxiety levels were accompanied by significant decreases in 5-HT^+^ neurons in medial PFC, ventral and lateral OFC superficial (II–III) and deep (V) layers, CA1 and CA3 hippocampal regions and BLA. In support, it has been found that vesicular monoamine transporter-deficient mice with significant reductions in NE and 5-HT levels in the striatum, hippocampus and cortex exhibited anxiety-like symptoms associated with PD [36].

Rodent prefrontal intermediate II/III and deep cell layers V/VI [37] consist of ~80% glutamatergic pyramidal projection neurons and ~20% GABAergic local interneurons. Serotonin and adrenergic receptors are mainly expressed in the pyramidal neurons of intermediate and deep layers of mPFC, modulating cortico-thalamic pathways [38]. Although we have not precisely documented the neuronal type of 5-HT, SERT and β_2_-ARs expressing cells in layers II/III and V, their size and morphology clearly suggest that they represent pyramidal cells. However, further studies are needed to determine whether serotonin and β_2_-ARs are co-localized, as suggested for α1-adrenoceptors and 5-HT2-R [39].

In the present study, decreased expression of SERT in the hippocampal regions and superficial layers of vOFC of rotenone-treated rats was coexistent with decreases in 5-HT^+^ cell densities, possibly representing a compensating mechanism to counteract decreased 5-HT levels. That is, SERT down-regulation could support limited 5-HT uptake, resulting in higher synaptic 5-HT levels, while decreasing cytoplasmatic levels. In agreement, 5-HT and SERT levels in striatum and PFC have been shown to decrease [40]. In addition, neuroimaging studies have shown a neocortical reduction in SERT density in animal models [41,42] and SERT down-regulation in the frontal cortices of patients with early PD without pharmacological treatment [43]. In contrast to the hippocampus and vOFC, decreased 5-HT immunoreactivity in BLA was accompanied by an increased SERT immunoreactivity, possibly leading to excessive clearance of synaptic serotonin further adding to the reduced levels of 5-HT. This could contribute to an exaggerated response of the fear circuit, resulting in anxiety-like behavior.

Interestingly, preclinical studies have shown correlations between depressive behavior and decreases in the hippocampal BDNF levels, as well as enhanced expression of BDNF following antidepressant treatment [44]. Indeed, double immunofluorescence of BDNF with SERT indicated the localization of the neurotrophic factor within a population of hippocampal serotoninergic cells. In vivo studies support that BDNF has a neuroprotective role in serotonergic neurons, for example, BDNF increased axon density in 5-HT neurotoxin lesioned rats [45]. Importantly, BDNF decreases in the dopaminergic nigrostriatal pathway were suggested to participate in the motor deficits of a rat rotenone PD-like model [21]. Overall, our data support that serotonergic deficits within the corticolimbic nodes may represent an underlying mechanism of the anxiety-like phenotype; however, further studies are necessary to determine the exact region-specific role of BDNF in animal PD models.

In addition, the PFC of rotenone-treated rats showed complex alterations in the expression of β_2_-ARs. Specifically, β_2_-AR expressing cells were decreased in vOFC, lOFC and hippocampal CA2 and CA3 in rotenone-treated rats, favoring the presence of a regulatory mechanism counteracting a possible increased noradrenaline release. These results are consistent with previous evidence demonstrating that elevated stress and norepinephrine levels are associated with decreased β_2_-AR expression in the hippocampus [46]. In contrast, β_2_-AR immunoreactivity was increased in BLA, probably reflecting the modulatory role of β_2_-ARs in amygdala, mediating the anxiety responses by increased amygdala activity in PD. A previous study, evaluating the monoaminergic dysregulation in corticolimbic domains of macaques, showed MPTP-induced NA depletion in the amygdala [47]. In agreement, reduction in noradrenaline concentration in the CSF of PD patients is significantly correlated with parkinsonian symptoms [48]. These data may indicate the significance of BLA region-specific β_2_ noradrenergic receptor up-regulation to the anxiety phenotype, possibly contributing to the decreased serotonergic neurotransmission in the rotenone PD model.

Currant supplementation, possibly due to its high phenolic content, reduced thigmotaxic behavior and partly improved the anxiety-like behavior induced by rotenone. Indeed, quantitatively determined phenolic content of Corinthian currants showed higher concentrations of isoquercetin, quercetin, kaempferol, resveratrol and rutin, as well as cinnamic and benzoic acid derivatives. Most of these were detected in the striatum, mesencephalon and hippocampus of rats fed a 3% Corinthian currant supplemented diet for 38 days [15,21]. In agreement with this, quercetin post-treatment of 6-OHDA injection resulted in a decrease in the apomorphine-induced rotational behavior [49]. Similarly, quercetin pre-treatment resulted in anti-inflammatory, antioxidant and neuroprotective actions, together with an improvement in motor performance [50,51]. In addition, resveratrol evoked antidepressant-like effects in chronic unpredictable mild stress model rats [52].

Importantly, parallel to the behavioral effects, currant supplementation significantly attenuated rotenone-induced BLA β_2_-AR increases and partly recovered serotonin cell density in deep layers of mPFC, vOFC, lOFC and BLA, possibly mediating anxiolytic-like effects. In agreement, using the tail-suspension test, a polyphenol rich intake has been previously established to exert anxiolytic-like effects in mice, possibly by increasing the availability of 5-HT and NA in the synaptic cleft [53]. Altogether, our study provides clear evidence of the beneficial effects of black Corinthian currants on the anxiety phenotype of rotenone-treated rats, possibly by rescuing 5-HT in corticolimbic areas.

## 4. Materials and Methods

### 4.1. Ethics Statement

The study was performed in accordance with the EU Directive 2010/63/EU for laboratory animal care and use and was approved by the Ethics committee of Patras University and by the Veterinary Administration of the Prefecture of Achaia, Greece (Protocol number: 187526/625/26-06-2018). All animal experiments were conducted and reported in accordance with ARRIVE guidelines and efforts were made to minimize animal suffering and to reduce the number of animals used.

### 4.2. Animals

Forty-eight adult male Wistar rats, weighing 250–300 g (postnatal days 60 to 70) were used. The rats were kept under standard conditions of temperature (22 ± 2 °C) and relative humidity (55 ± 5%) with a 12-light/12-dark cycle. Rats were handled for a 10-day period to be familiarized with the researchers.

### 4.3. Experimental Design

Rotenone (Sigma, St. Louis, MO, USA) was suspended in vehicle solution containing 1% dimethylsulfoxide (Sigma, St. Louis, MO, USA) in sunflower oil and injected subcutaneously (s.c.) in a volume of 2.5 mg/kg of body weight daily for 28 days [54]. Rotenone was vortexed thoroughly just before injection to ensure a uniform suspension. Currant supplementation was provided as independent food and the determination of daily consumption was based on previous evidence [21]. Forty-eight rats were randomly divided into 4 groups, each containing 12 rats as follows:

Control (CTR): Vehicle-injected rats (1 mL/kg/day, s.c.) containing 1% dimethylsulfoxide in sunflower oil for 28 days, following 10 days handling.

Rotenone (ROT): Rotenone-treated rats (2.5 mg/kg/day, s.c.) for 28 days to induce experimental PD-like phenotype, following 10 days handling.

Control_currant (CTR_CUR): Currant supplemented diet (3% of their daily food intake) for 38 days starting 10 days prior to vehicle injections (1 mL/kg/day, s.c., 28 days).

Rotenone_currant (ROT_CUR): Currant supplemented diet (3% of their daily food intake) for 38 days, starting 10 days prior to rotenone treatment (2.5 mg/kg/day, s.c., 28 days).

### 4.4. Behavioral Testing

**Figure 8:**
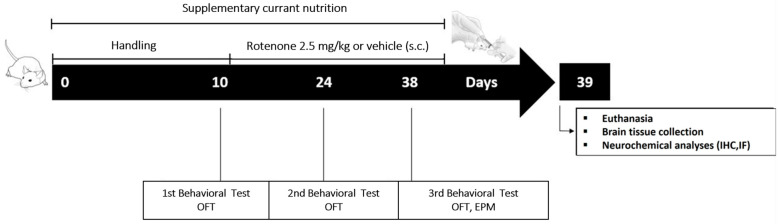
Experimental protocol and time points of the behavioral testing and neurochemical analyses. Schematic representation showing the experimental design and endpoints measured to analyze rat anxiety-like phenotype. OFT: Open Field Test, EPM: Elevated Plus Maze test, s.c.: subcutaneously.

Rats were tested in a battery of tasks to determine exploratory and anxiety-like behavior. Prior to behavioral tests, the subjects were allowed to adjust to the new conditions of the testing room for 2 h. Behavior was recorded using a video-tracking system and quantified with the Noldus Ethovision XT7 software. The experimental protocol is shown in Figure 8.

#### 4.4.1. Open Field

The OFT was used to estimate rat thigmotaxic behavior, i.e., the tendency to stay close to the wall. Rats were placed in a clear Plexiglas open field (100 × 100 × 50 cm) and a digital video camera (SONY/HDR-CX625) was installed above the apparatus. On days 10 (2 h after the first injection), 24 and 38 rats were individually placed in the center of the open field apparatus and allowed to explore the field for 10 min.

#### 4.4.2. Elevated Plus Maze

The EPM test consisted of two opposite open (50 cm long × 10 cm wide) and two enclosed arms (50 × 10 × 40 cm) which emerged from a central platform (10 × 10 cm) elevated 50 cm above floor level, based on a design validated by Lister [55].

At the end of the experiment, 1 h after the OFT, each rat was placed in the center of the apparatus facing a closed arm and allowed to explore the maze for 5 min. The time spent on each arm and the latency to the first entry in open arms were recorded. The anxiety index, an index that integrates the EPM behavioral measures, was calculated as follows: anxiety index = 1 − [(Open arm time/Test duration) + (Open arms entries/Total number of entries)/2]. The anxiety index values range from 0 to 1 where an increase in the index expresses increased anxiety-like behavior [56]. The surface of the EPM was cleaned with 70% alcohol before each test.

### 4.5. Immunohistochemistry: Immunofluorescence

On day 39, rats were anaesthetized with a dose of ketamine (100 mg/kg) and xylazine (10 mg/kg), and transcardially perfused with 0.9% NaCl and ice-cold paraformaldehyde (4%) in 100 mM phosphate-buffered saline (PBS), pH 7.4. Brains were removed, cryoprotected in two successive baths of 15% and 30% sucrose/PBS at 4 °C, embedded in tissue-freezing medium (Jung, Leica Instruments, Instr., Wetzlar Germany) and frozen in 2-methyl-butane. Coronal sections, 20 μm thick, were cut in a cryostat Leica, thaw-mounted on gelatin–chromalum-coated glass slides, dried at RT and stored at 75 °C.

Single and double labeling immunohistochemical analyses were performed as previously described [57]. For single-labeling experiments, before immunostaining, antigen retrieval in citrate buffer at 85 °C for 20 min and blocking of endogenous peroxidase with 0.03% H_2_O_2_ were performed. Following serum blocking (1% NHS with 5% BSA, Sigma–Aldrich, Deisenhofen, Germany and 0.5% Triton X-100 in PBS) for 60 min at RT, sections were incubated overnight at 4 °C with rabbit anti-5-HT (1:700, overnight, Sigma Aldrich, Deisenhofen, Germany), mouse anti-SERT (1:100, overnight, Santa Cruz, TX, USA) or mouse anti-β_2_-AR (1:100, for 40 h, Santa Cruz, TX, USA) diluted in 0.01M PBS with 0.5% Triton X-100 (PBST) with 1% BSA and 0.15% NHS at 5–6 °C. Sections were then incubated in biotinylated donkey anti-rabbit secondary antibody for 50 min (for 5-HT) and biotinylated horse anti-mouse IgG (1:200 for 2 h) (for SERT and β_2_-ARs) at RT. Slides were treated for 1 h in the dark with the ABC Vectastain kit (Vector Laboratories, Newark, CA, USA) with diaminobenzidine (DAB, Vector Laboratories, Newark, CA, USA) used as the chromogen. After rinsing with cold PBS buffer, dehydrating with alcohols and clearing with xylenes, slides were coverslipped with Entellan (Merck, Darmstadt, Germany).

Immunofluorescence experiments were performed to determine the expression pattern of 5-HT expressing cells and fibers. Briefly, following blocking, sections were incubated overnight at 4 °C with rabbit anti-5-HT (1:400). After washing with PBS three times, the sections were incubated for 2.5 h at room temperature with Alexa-488 conjugated donkey anti-rabbit IgG (1:400, Molecular Probes, Leiden, the Netherlands) in 0.5% PBS-T. Slices were washed five times in PBS for 10 min each and were counterstained with 4′,6-diamidino-2-phenylindole (DAPI, Molecular Probes, Leiden, the Netherlands).

Double immunofluorescence experiments were performed to determine the co-localization of BDNF on SERT expressing cells and fibers. Briefly, following blocking, sections were incubated with a mixture of primary antibodies diluted in blocking solution for 40 h at 6–7 °C. Antibodies used included monoclonal mouse anti-SERT (1:50) and polyclonal rabbit anti-BDNF (1:300, Novus Biologicals, Englewood, CO, USA). Then, sections were washed in PBS three times, followed by incubation with designated secondary antibodies (Alexa-555 conjugated donkey anti-mouse IgG and Alexa-488 conjugated donkey anti-rabbit IgG (1:400), Molecular Probes, Leiden, the Netherlands) in 0.5% PBS-T for 2.5 h. Slices were washed five times in PBS for 10 min each and coverslipped with fluorescent hard medium (H-1400, Vector Laboratories, Newark, CA, USA).

Details on the antibodies used are shown in Supplementary Table S1.

### 4.6. Quantifications and Photomicrograph Production

Single immunofluorescent images were captured using a colored digital camera CFW-1308C (Scion Corp., Chicago, IL, USA) attached to a Nikon Eclipse E800 optical and fluorescent microscope (Nikon, Tokyo, Japan) and connected to a PC. Double immunofluorescence images were captured using a Leica SP8 confocal microscope. Microscopic images from immunohistochemistry experiments were captured using the Nikon/Optiphot 2 microscope connected to a PC via a color CCD SONY camera (DXC-950P) at X100 and X400 magnification. The immunostained cell bodies for either 5-HT, SERT or β2-ARs were analyzed manually in higher magnification images of PFC, hippocampus and basolateral amygdala. Specifically, cell densities were determined within orbitofrontal and medial prefrontal cortical layers II/III and V. Positive neuronal cell bodies were quantified in three sections (distances of 100 μm) per region per animal, using a graduated frame of 100 μm^2^ (×3 graduated frames per section). The nomenclature used was based on the topological rat brain atlas of Paxinos and Watson [24].

### 4.7. Statistical Analysis

The statistical program SPSS (SPSS Statistics 27.0) was employed throughout. Statistical analysis for OFT was evaluated using two-way repeated-measures ANOVA followed by an independent *t*-Test. For EPM behavioral tests and neurochemical studies, the analysis was performed using a two-way analysis of variance (ANOVA) followed by an independent t-Test. A probability level of 5% (*p* < 0.05) was considered statistically significant. Data were expressed as mean ± standard error of measurement (SEM) and graphs were constructed in GraphPad Prism 6.0 (GraphPad Software Inc., San Diego, CA, USA). Correlation analysis was evaluated by Pearson product-moment correlation analysis in SPSS and visualized using Origin 2022 (OriginLab Corporation, Northampton, MA, USA).

## 5. Conclusions

Rotenone-treated rats exhibited increased anxiety levels, in agreement with the depression and anxiety symptoms in PD patients. Our results revealed complex changes in adrenergic and serotonergic transmissions across the corticolimbic circuit, accompanying the behavioral phenotype. Supplementation with black Corinthian currants, a dried fruit rich in polyphenols, attenuated rotenone-induced anxiety-like behavior and partly reversed the rotenone-induced alterations in the monoaminergic markers studied. Taken all together, the regulation of 5-HT, SERT and β_2_-ARs expression in PFC and BLA may underly the alleviated rotenone-induced anxiety-like behaviors caused by currant consumption. Future research is needed to better understand the specific role of serotonergic transmission in PFC and BLA in anxiety and to determine the underlying mechanisms mediating the polyphenols’ neuroprotective actions in serotonergic transmission in PD.

## Data Availability

The raw data supporting the data presented in this study are readily available on request from the corresponding author.

## Supplementary Materials

The following supporting information can be downloaded at: https://www.mdpi.com/article/10.3390/ijms24010462/s1.
