# Supplementary material for: Protective Effects of Currants (Vitis vinifera) on Corticolimbic Serotoninergic Alterations and Anxiety-like Comorbidity in a Rat Model of Parkinson’s Disease
Source: Int J Mol Sci. 2022 Dec 27;24(1):462. doi: 10.3390/ijms24010462 (PMC9820698; doi:10.3390/ijms24010462)
Supplement: Supplementary file 1 [file ijms-24-00462-s001.zip › ijms-2090722-SI.pdf]

Table S1: Primary and Secondary antibodies used.

**Primary**	**Host**	**Source**	**Application**	**Dilution**
β2-AR<br>sc-81577	Mouse	Santa Cruz, Texas U.S.A.	IHC	1:100(IHC)
5-HT<br>S5545	Rabbit	Sigma Aldrich, Deisenhofen, Germany	IHC<br>IF	1:700 (IHC)<br>1:400(IF)
SERT<br>sc-518084	Mouse	Santa Cruz	IHC<br>IF	1:100 (IHC)<br>1:50(IF)
BDNF<br>NB-100-98682	Rabbit	Novus Biologicals Englewood, Colorado, USA	IF	1:300 (IF)
Secondary				
Mouse IgG	Horse	Vector Laboratories, Newark, CA, USA	IHC	1:200
Rabbit IgG	Horse	Vector	IHC	R.T.U.
Mouse IgG-555	Donkey	Molecular probes, Leiden, the Netherlands	IF	1:400
Rabbit IgG-488	Donkey	Molecular probes	IF	1:400

Note: *To test the specificity of the primary antibodies used, additional negative controls were performed in adjacent sections, with the omission of the primary antibody or with application of secondary antisera mismatched for species. No labeling was observed in any case.*

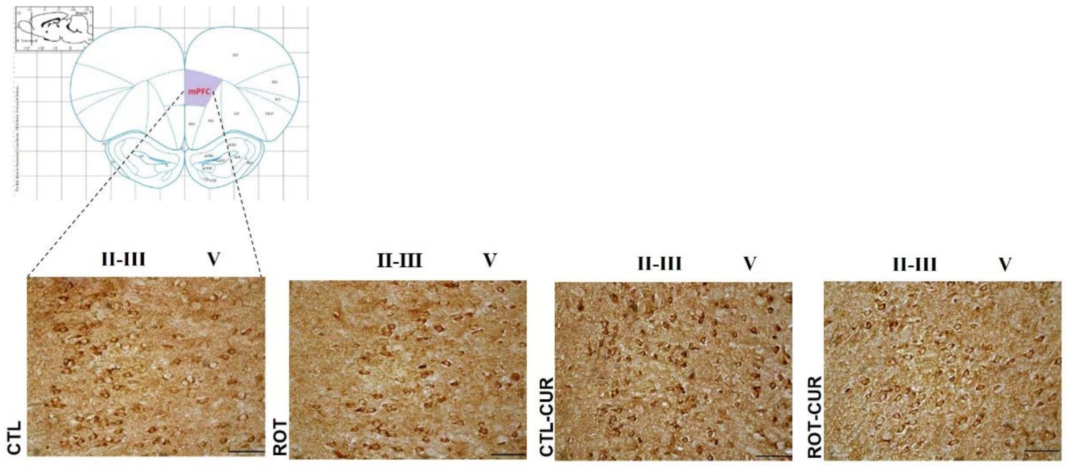

Figure S1: Representative microphotographs showing 5-HT immunostaining in mPFC at the coronal level indicated in the schematic diagram from Paxinos and Watson [24]

rat brain atlas, in a rotenone–induced PD model with or without currant consumption Scale bar=0.05mm.

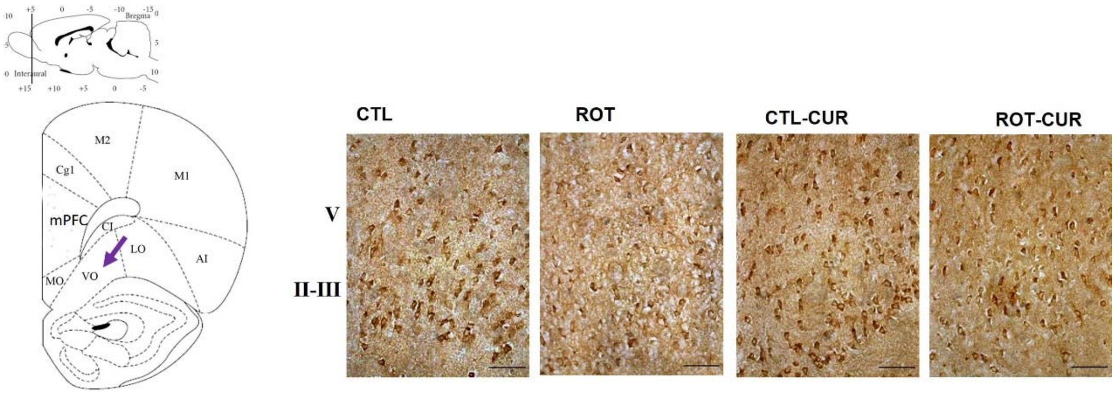

Figure S2: Representative microphotographs showing 5-HT immunostaining in vOFC sections at the coronal level of the vOFC (VO) indicated in the left (from Paxinos and Watson [24] rat brain atlas) in a rotenone–induced PD model with or without currant consumption Scale bar=0.05mm.

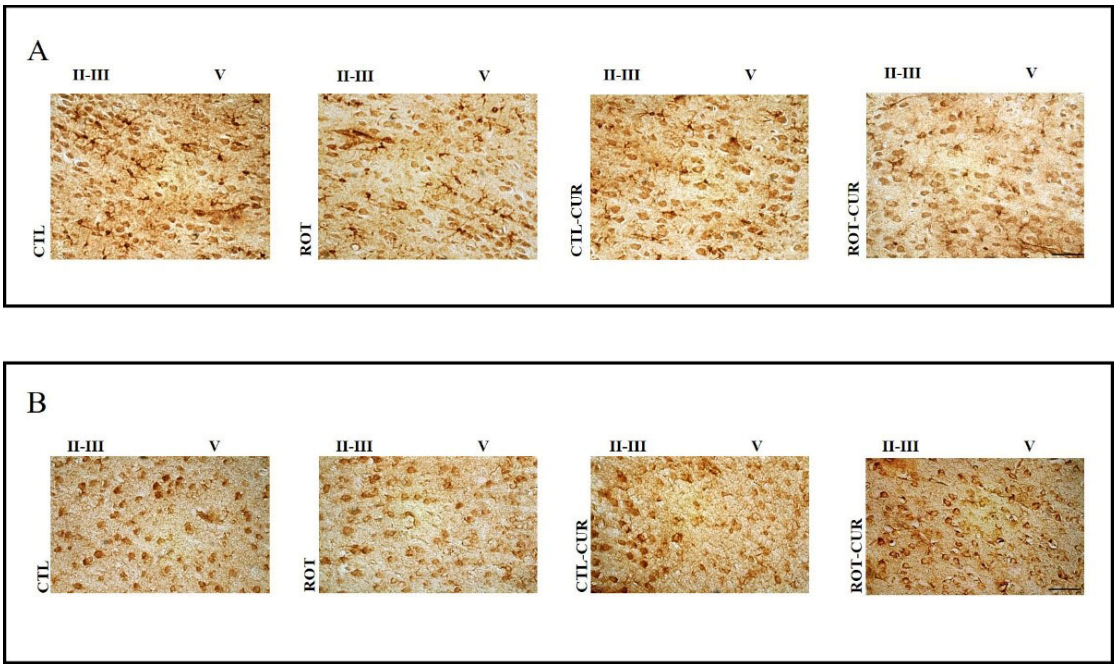

Figure S3: Representative microphotographs showing mPFC sections immunostained for (A) SERT and (B) β_2-ARs in a rotenone–induced PD model with or without currant consumption. Scale bar=0.05mm.
